# Histopathology of Incontinence-Associated Skin Lesions: Inner Tissue Damage Due to Invasion of Proteolytic Enzymes and Bacteria in Macerated Rat Skin

**DOI:** 10.1371/journal.pone.0138117

**Published:** 2015-09-25

**Authors:** Yuko Mugita, Takeo Minematsu, Lijuan Huang, Gojiro Nakagami, Chihiro Kishi, Yoshie Ichikawa, Takashi Nagase, Makoto Oe, Hiroshi Noguchi, Taketoshi Mori, Masatoshi Abe, Junko Sugama, Hiromi Sanada

**Affiliations:** 1 Department of Gerontological Nursing/Wound Care Management, Graduate School of Medicine, The University of Tokyo, Tokyo, Japan; 2 Research fellow of Japan Society for the Promotion of Science, Tokyo, Japan; 3 Research Institute for Biomedical Sciences, Tokyo University of Science, Chiba, Japan; 4 School of Nursing, University of Shizuoka, Shizuoka, Japan; 5 Department of Advanced Nursing Technology, Graduate School of Medicine, The University of Tokyo, Tokyo, Japan; 6 Department of Life Support Technology (Molten), Graduate School of Medicine, The University of Tokyo, Tokyo, Japan; 7 Sapporo Skin Clinic, Kojinkai, Sapporo, Hokkaido, Japan; 8 Department of Clinical Nursing, Graduate School of Medical Science, Kanazawa University, Ishikawa, Japan; University Hospital Hamburg-Eppendorf, GERMANY

## Abstract

A common complication in patients with incontinence is perineal skin lesions, which are recognized as a form of dermatitis. In these patients, perineal skin is exposed to digestive enzymes and intestinal bacterial flora, as well as excessive water. The relative contributions of digestive enzymes and intestinal bacterial flora to skin lesion formation have not been fully shown. This study was conducted to reveal the process of histopathological changes caused by proteases and bacterial inoculation in skin maceration. For skin maceration, agarose gel containing proteases was applied to the dorsal skin of male Sprague-Dawley rats for 4 h, followed by *Pseudomonas aeruginosa* inoculation for 30 min. Macroscopic changes, histological changes, bacterial distribution, inflammatory response, and keratinocyte proliferation and differentiation were examined. Proteases induced digestion in the prickle cell layer of the epidermis, and slight bleeding in the papillary dermis and around hair follicles in the macerated skin without macroscopic evidence of erosion. Bacterial inoculation of the skin macerated by proteolytic solution resulted in the formation of bacteria-rich clusters comprising numerous microorganisms and inflammatory cells within the papillary dermis, with remarkable tissue damage around the clusters. Tissue damage expanded by day 2. On day 3, the proliferative keratinocyte layer was elongated from the bulge region of the hair follicles. Application of proteases and *P*. *aeruginosa* induced skin lesion formation internally without macroscopic erosion of the overhydrated area, suggesting that the histopathology might be different from regular dermatitis. The healing process of this lesion is similar to transepidermal elimination.

## Introduction

A common complication in patients with incontinence is the formation of skin lesions in the perineal area due to skin maceration [1,2]. It has been reported that the prevalence of urinary and/or fecal incontinence among nursing home residents is 59.8% [3]. The elderly with incontinence are at substantial risk for cutaneous disorders, as demonstrated by the presence of skin lesions in 36.0% of older patients in Japanese long-term medical facilities [4].

Perineal skin lesions are often observed among patients with fecal incontinence. Bliss et al. [3] examined the correlation between incontinence and perineal dermatitis in nursing home residents. They concluded that fecal and double (both urinary and fecal) incontinence significantly correlated with perineal dermatitis. Their research indicates that fecal incontinence is a crucial factor in the development of skin lesions.

In the healthcare setting, several interventions are implemented for preventing skin lesions induced by fecal incontinence. However, Driver [5] reported that perineal skin lesions still developed with an incidence of 19% in critical care unit patients with fecal incontinence, even when skin care using a one-step cleaning and protectant product was provided for 4 weeks. These skin lesions are accompanied by tingling, itching, burning, and pain [2], which negatively influence patient quality of life [6]. Complete prevention of perineal skin lesions due to fecal incontinence is a pressing nursing issue.

Although incontinence-associated skin lesions are usually diagnosed as contact dermatitis, their histopathology has not been fully shown. The process of skin lesion formation due to incontinence is thought to include two steps: (1) skin maceration, and (2) exposure to fecal components. Skin maceration, which is caused by exposure to excessive water, has been recognized as one of the risk factors for skin lesions [1,2,7]. Skin maceration disrupts the intercellular lipid lamellae in the corneal layer of the epidermis [8–11], thereby inducing increased transepidermal water loss (TEWL) [9,12] and transdermal penetration of macromolecules [9,10]. Moreover, expansion of the interstitial space and a decreased number of cell processes have also been observed in the basal and prickle cell layers of the epidermis [9]. Based on these results, Minematsu et al. [13] defined skin maceration as a functional disorder of the barrier to irritants and tolerance for force due to structural alteration of intercellular lipid layers and junctions between keratinocytes in the epidermis. Inflammatory response due to invasion of irritants and vulnerability due to decreased intercellular junction are recognized as triggers for the formation of skin lesions.

Previously, researchers have focused on the effect of exposure to excessive water in skin lesions. However, given the role of fecal incontinence in the development of perineal skin lesions, we considered whether the effects of digestive enzymes and intestinal bacterial flora are also important factors. Feces contains several digestive enzymes, including trypsin, α-chymotrypsin, and lipase [14,15]. In particular, it has been reported that proteases enhance the transdermal penetration of macromolecules [16,17], suggesting that proteases themselves can invade and digest skin tissue. In our study, protease-associated skin maceration is termed “proteolytic skin maceration” to emphasize its role in the development of incontinence-associated skin lesions. Until now, the effects of proteolytic skin maceration on the tissue structure of skin have not been studied.

Feces contain many intestinal bacteria, over 10^11^ bacterial cells per gram of feces [18,19]. In patients with fecal incontinence, the perineal skin is frequently exposed to high-density bacterial flora. Bacterial inoculation of murine skin pretreated by tape stripping, known to be a barrier-impairing technique with minimal tissue damage, has been shown to induce crust formation or epidermal proliferation [20]. Although these results suggest that tissue damage is probably due to the virulence of inoculated bacteria in skin with impaired barrier function similar to that caused by proteolytic skin maceration, the contribution of fecal bacterial flora to the maceration-associated skin lesions has not been studied.

To prevent incontinence-associated skin lesions, advanced skin care that addresses fecal components including digestive proteases and bacterial load is essential. This study was conducted to reveal the process of histopathological change caused by proteases and bacterial inoculation in skin maceration. Our findings indicated a difference in histopathology and manner of healing between incontinence-associated skin lesion and skin lesion from contact dermatitis.

## Materials and Methods

### Chemicals

Bovine serum albumin (BSA), trypsin from porcine pancreas, α-chymotrypsin from bovine pancreas, and histofine were purchased from Nacalai Tesque (Kyoto, Japan). Carboxyfluorescein diacetate succinimidyl ester (CFSE) was from Invitrogen (Carlsbad, CA). Pentobarbital sodium was from Kyoritsu Seiyaku (Tokyo, Japan). Biotin-conjugated anti-*Pseudomonas* species polyclonal antibody, anti-MHC-II monoclonal antibody (OX-6) and anti-keratin 10 mouse monoclonal antibody (DE-K10) were from Thermo Fisher Scientific (Waltham, MA). Anti-Ki 67 mouse monoclonal antibody (MIB-5) was from Dako (Glostrup, Denmark). VectaStain ABC Kit and biotin-conjugated anti-mouse IgG antibody were from Vector Laboratories (Burlingame, CA), and 3,3′-diaminobenzidine tetrahydrochloride (DAB Tablet) was purchased from Wako Pure Chemical (Osaka, Japan).

### Animals

Seventeen 6-month-old male Sprague-Dawley rats (Japan SLC, Shizuoka, Japan) were bred in specific pathogen-free animal facilities. Animal experiments were performed according to the National Institutes of Health (NIH) Guide for the Care and Use of Laboratory Animals and were approved by the Animal Research Committee of The University of Tokyo (Permit number: P10-141). All animal procedures were performed under sodium pentobarbital anesthesia to avoid causing animal discomfort.

### Proteolytic solution exposure

A modified skin maceration model was used for reproduction of proteolytic skin maceration [9]. Briefly, agarose gels containing proteases were applied to dorsal skin. Dorsal skin was selected as the treatment area to minimize contact from urine or feces excreted from the host.

Approximately 100 μL droplets of 1% agarose gel dissolved in 153 mM Tris-HCl buffer (pH 7.4) were immersed in 153 mM Tris-HCl buffer (pH 7.4) or proteolytic solution (0.25% *wt*/*vol* trypsin, 0.40% *wt*/*vol* α-chymotrypsin in Tris-HCl buffer) and gently shaken overnight in a cooler box. The concentrations of both trypsin and α-chymotrypsin in the proteolytic solution were determined according to the previous report [15]. Dorsal hair of rats was removed by a shaver and depilatory cream under anesthesia with pentobarbital sodium (30 mg/kg-40 mg/kg, intraperitoneally) 3 days before treatment in order to minimize the effect of depilatory cream on skin. Skin maceration treatment (MT, n = 4) or proteolytic treatment (PT, n = 5) were performed by applying one agarose gel droplet to one side of the dorsal skin (between the bottom of the shoulder blade and the top of the femur) in each rat using a polyurethane film dressing (Tegaderm transparent dressing; 3M, St. Paul, MN) for 4 h. No treatment (NT) was applied to the contralateral side of the skin. After removal of the agarose gel, the skin was air-dried for 30 min, and skin hydration (Moisture Checker; Scalar, Tokyo, Japan) and TEWL (VapoMeter; Delfin Technologies, Kupio, Finland) were measured on both the PT and corresponding NT area. The appearance of the skin surface was recorded using a digital camera (LUMIX DMC-FX-60; Panasonic, Tokyo, Japan).

### Bacterial inoculation

Bacterial inoculation was performed with *Pseudomonas aeruginosa* (PAO1) which is one of the bacteria indigenous to the intestinal tract and skin and is detected in perineal skin in some patients with incontinence [21]. *P*. *aeruginosa* was selected as a model because of the importance of bacterial motility, as suggested by preliminary tests in which no transdermal penetration of insoluble particles (diameter, 50 nm to 500 nm) was observed in PT skin. Most of the major intestinal bacteria, such as *Escherichia coli* or *Clostridium difficile*, have one or more of types of motility patterns, including swimming, swarming, and twitching [22]. Therefore, a bacterial strain such as *P*. *aeruginosa*, which demonstrates multiple types of motility patterns, was considered a suitable model for intestinal bacterial in these experiments [23, 24].

Bacteria cells were cultured overnight in 6 mL of Luria-Bertani broth at 37°C on a shaker, centrifuged at 1,700 ×*g* for 15 min, and resuspended in phosphate-buffered saline at a concentration of OD_600_ = 0.6, equivalent to 2.5 × 10^9^ CFU/mL. The bacterial concentration was determined by preliminary testing and was lower than the previously reported concentration in human feces [18, 19]. The suspension was labelled with 10 μM CFSE for 30 min at 37°C in the dark. A pair of round filter papers (diameter, 8 mm) were soaked with CFSE-labelled bacterial suspension (25 μL each) and applied to the PT and NT skins for 30 min (PT-B and NT-B, respectively, n = 5).

### Histological and immunohistological analyses

The collected tissue samples were immediately divided into two pieces and fixed with 4% paraformaldehyde in phosphate buffer (pH 7.4) and then embedded in paraffin or OCT compound (Sakura Finetek, Tokyo, Japan).

Approximately 4 μm-thick paraffin sections were used for histological analysis by hematoxylin and eosin (HE) staining and by immunohistochemistry for MHC-II, Ki 67, keratin 10 and *Pseudomonas* species. For immunohistochemistry, endogenous peroxidase activity in sections was inactivated by incubation in 0.3% hydrogen peroxide/methanol for 30 min. Antigen retrieval by autoclaving at 121°C for 15 min in 10 mM citrate buffer (pH 6.0) was required for MHC-II and Ki 67. The immunoactivity of antibodies for MHC-II (diluted 1:200, 60 min) was detected by the biotin-conjugated anti-mouse IgG antibody (diluted 1:1000, 30 min). Bacterial localization was detected with biotin-conjugated anti-*Pseudomonas* antibody (diluted 1:300, 60 min). The immunoactivity of antibodies for Ki 67 (diluted 1:300) and keratin 10 (diluted 1:600) were detected using a polymerized secondary antibody (histofine). The immunoreactions were enhanced by ABC method for MHC II and *Pseudomonas* species, visualized with DAB, and counterstained with hematoxylin.

Bacterial colonization on day 1 was also identified by the observation of fluorescent signal of CFSE on approximately 5 μm-thick frozen sections that were counterstained with 4',6-diamidino-2-phenylindole (DAPI). The observation of sections was performed using an inverted fluorescence microscope (Leica DMI 4000B; LEICA, Wetzlar, Germany).

### Statistical analysis

For analysis of skin hydration and TEWL, the mean value of three stable measurements was used. The difference in values between PT, MT and NT group was analyzed by one-way ANOVA. Data are reported as means ± standard deviation.

## Results

### Proteolytic skin maceration model

To validate that skin maceration was reproduced by our method, skin hydration and TEWL were measured. These measurements were performed after 30 min of air-drying following exposure to proteolytic solution in order to avoid possible measurement failure when the skin surface was wet [25,26]. Compared with MT and NT skin, exposure to proteolytic solution increased levels of skin hydration (PT: 29.9% ± 3.4%; MT: 22.2% ± 4.8%; NT: 15.9% ± 5.4%, *p* < 0.001) and TEWL (PT: 55.9 g/m^2^h ± 36.9 g/m^2^h; MT: 33.8 g/m2h ± 9.9 g/m2h; NT: 11.9 g/m2h ± 5.1 g/m2h, *p* = 0.001). These results indicate skin maceration causes overhydration and impairment of skin barrier function, and that these effects are accelerated by proteases treatment.

### Histological change in proteolytic skin maceration

To reveal the effects of proteolytic skin maceration, we compared macroscopic skin appearance, histological changes, and inflammatory response between the PT and NT groups in five each rats.

**Fig 1:**
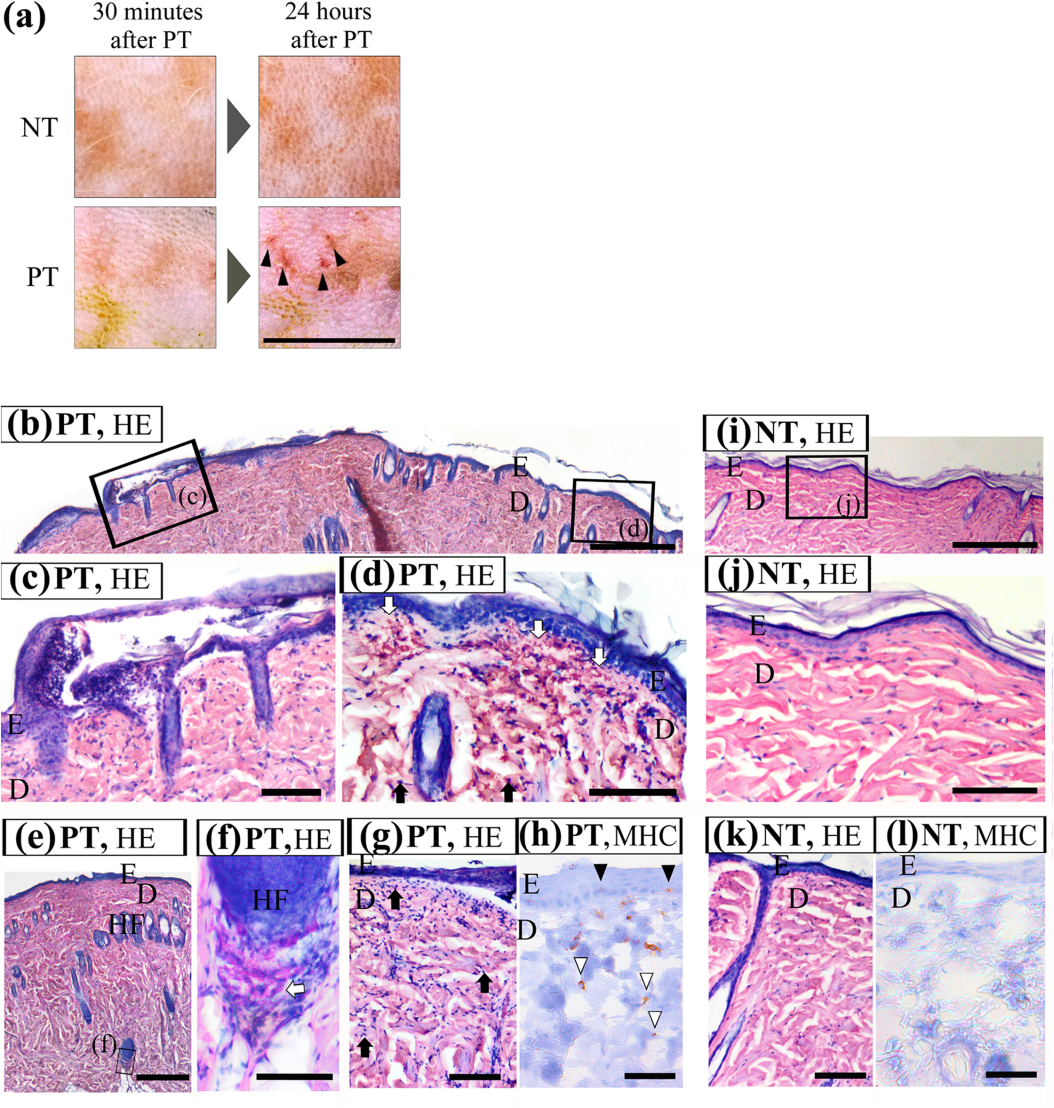
Comparison of proteolytic skin maceration and no treatment skin. (a) Macroscopic skin appearance of proteolytic skin maceration. Skin appearance was observed at both 30 min (left side) and 24 h (right side) after removal of agarose gels. The upper half of both panels is the macerated area. At 24 h after treatment, flare and dot-like redness (black arrowheads) were observed in PT skin. No erosion was observed in either group. Scale bar = 1 cm. (b-j) Histological change and inflammatory response in proteolytic skin maceration. Histological structure and inflammatory response were observed in NT skin (i-l) and in contralateral proteolytic macerated skin (PT; b-h) 24 h after treatment. Thin sections were stained with HE (b-g, i-k) and with immunohistochemistry for MHC-II (h, l). Two boxed fields in (b) are magnified in (c) and (d). The boxed field in (i) is magnified in (j). Proteolytic skin maceration induced epidermal digestion in the prickle cell layer (c), infiltration of inflammatory cells (d and g, black arrows) in the papillary and reticular dermis. Slight leakages of erythrocytes (d and f, white arrows) were observed in the papillary dermis and around hair follicles in PT skin. MHC-II–positive cells detected by DAB staining distributed from the prickle cell layer of the epidermis (h, black arrowheads) to the reticular layer of the dermis (h, white arrowheads) in PT skin. Scale bar = 500 μm in (b), (e) and (i), and 100 μm in (c), (d), (f)-(h), and (j)-(l); E, epidermis; D, dermis; HF, hair follicle.

Fig 1A shows macroscopic skin appearance. At 30 min after treatment, no macroscopic change of skin was evident in the NT group, whereas PT skin became soft and turned whitish in appearance. At 24 h after treatment, flare and dot-like redness were observed in four of the five PT skins, which resembled punctate bleeding. No erosion was observed in either group.

HE and immunohistochemical staining for MHC-II are shown in Fig 1B–1H (PT group) and Fig 1I–1L (NT group). No abnormalities were observed in NT sections (Fig 1I and 1J). In contrast, digestion of the epidermal prickle cell layer (Fig 1B and 1C) and slight bleeding in the papillary dermis (Fig 1B and 1D) and around hair follicles (Fig 1E and 1F) were observed in PT sections. Infiltration of numerous inflammatory cells in the papillary and reticular dermis was also observed in PT sections (Fig 1G). Some of these inflammatory cells were identified by MHC-II immunoreaction as antigen-presenting cells (APCs), including epidermal Langerhans cells and dermal macrophages (Fig 1H). In contrast, inflammatory cells were rarely observed in NT sections (Fig 1K and 1L). These results indicate degradation of skin inner tissue and inflammatory response to foreign antigens and protease-digestives, and reflect macroscopic observations (flare and dot-like redness) in PT skin.

### Effect of bacterial inoculation

To reveal the effects of proteolytic skin maceration on bacterial invasion, we compared macroscopic skin appearance, histological changes, inflammatory response, and bacterial localization between PT-B and NT-B groups, each containing 5 rats.

**Fig 2:**
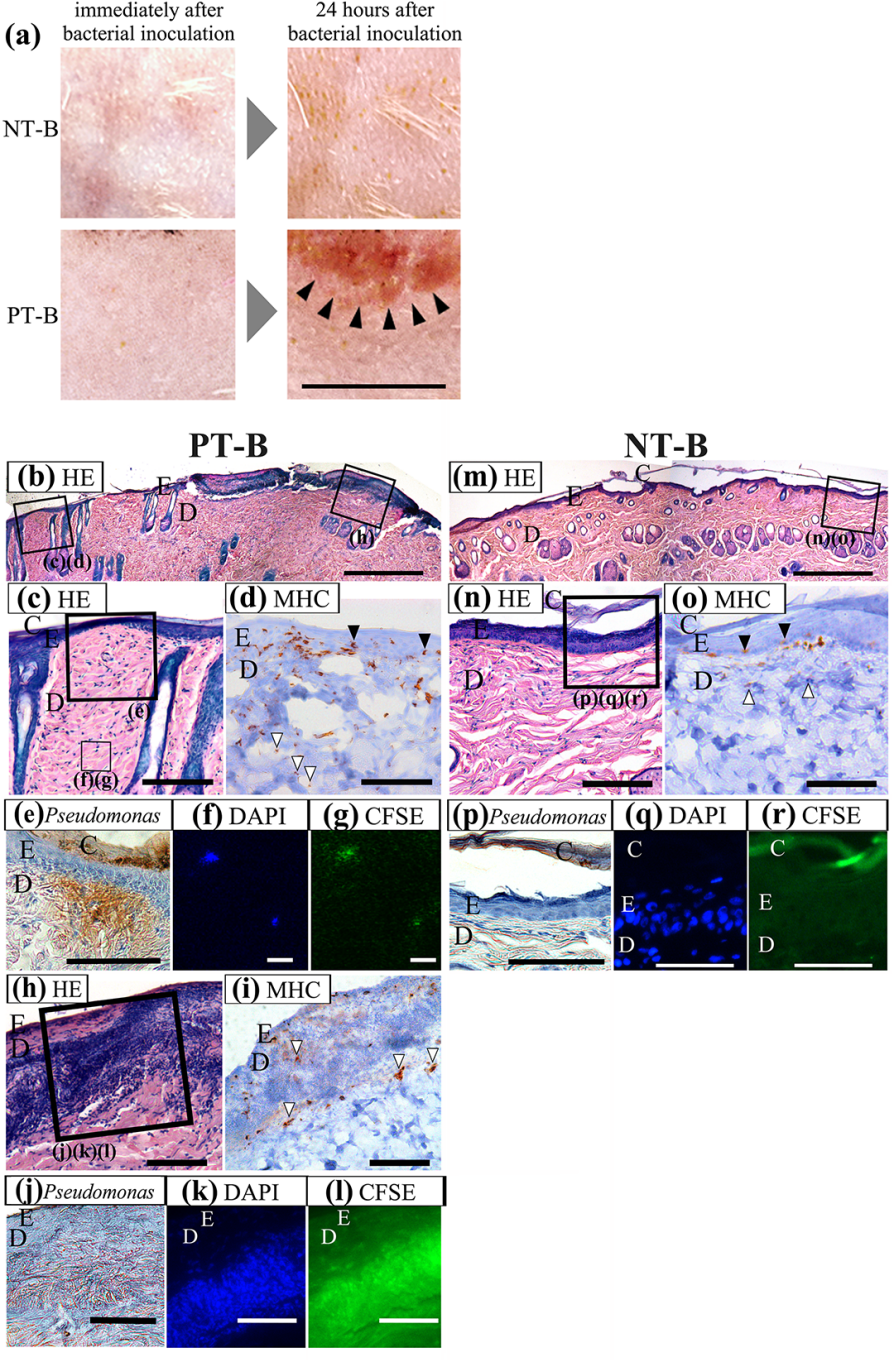
The effect of bacterial inoculation on proteolytic skin maceration and on no treatment skin. (a) Macroscopic skin appearance of proteolytic skin maceration inoculated with bacteria. Skin appearance was observed immediately after removal of filter paper soaked with bacterial suspension (left side), and also at 24 h after bacterial inoculation (right side). The upper half of each photograph is the treatment area. Immediately after treatment, no macroscopic skin abnormality was observed in either group. At 24 h after bacterial inoculation, the macroscopic flare expanded in the PT-B group only (black arrowheads). No erosion was observed in either group. Scale bar = 1 cm. Histological change and bacterial localization were observed in proteolytic macerated skin inoculated with fluorescence-labelled bacteria (PT-B; b-l) and non-macerated skin (NT-B; m-r). Tissue samples were collected 24 h after bacterial inoculation. Paraffin sections were stained with HE (b, c, h, m, n) and with immunohistochemistry for MHC-II (d, i, o) and for *Pseudomonas* species (e, j, p). The frozen sections were counterstained with DAPI (f, k, q), and CFSE signals of bacteria (g, l, r) were observed. Two boxed fields in (b) are magnified to (c), (d) and (h). The boxed field in (m) is magnified to (n) and (o). The larger and smaller boxed fields in (c) are magnified in (e) and (f), (g), respectively. The boxed area in (h) is magnified in (j)-(l). The boxed area in (n) is magnified in (p)-(r). Although no structural alteration was observed in NT-B samples (m, n), the numbers of inflammatory cells and MHC-II–positive cells were increased in the prickle cell layer of the epidermis (o, black arrowheads) and the papillary layer of the dermis (o, white arrowheads). The bacteria localized only in the corneal and granular layer of epidermis. In PT-B samples, bacterial cluster formation was observed in the epidermis and papillary dermis (h). Infiltration of inflammatory cells and MHC-II–positive cells was expanded to the deep layer of the reticular dermis (d, white arrowheads). The distribution of bacteria was frequently observed in the papillary dermis and rarely observed in the reticular dermis. In all PT-B samples, cellular clusters comprising bacteria and inflammatory cells were observed in the papillary dermis (i, white arrowheads). Scale bar = 500 μm in (b) and (m), and 100μm in (c)-(e), (h)-(l) and (n)-(r), and 10 μm in (f) and (g); C, corneal layer; E, epidermis; D, dermis.

Fig 2A shows macroscopic skin appearance. Immediately after bacterial inoculation, no macroscopic skin abnormality was observed in either group. At 24 h after bacterial inoculation, the macroscopic flare expanded in the PT-B group only. No erosion was observed in either group.

Histology and the distribution of APCs and bacteria are shown in Fig 2B–2L (PT-B group) and Fig 2M–2R (NT-B group). In the NT-B group, no histological alteration was identified (Fig 2M and 2N). In the PT-B group, however, remarkable infiltration of inflammatory cells in the papillary and reticular dermis was observed (Fig 2B and 2C). APC distribution was observed in the basal cell layer of the epidermis and the papillary layer of the dermis in the NT-B group (Fig 2O), whereas it expanded to the deeper layer of the reticular dermis in the PT-B group (Fig 2D). The APC distribution was observed in a deeper layer and more frequently in PT-B skin compared with PT skin. These results suggested the penetration and expansion of planktonic bacteria to the dermal layer in PT-B skin. In NT-B skin, *P*. *aeruginosa* was localized only in the corneal and granular layers of the epidermis (Fig 2P–2R), suggesting the blockage of bacterial invasion into the skin by barrier function. In contrast, immunoreactivity to *P*. *aeruginosa* in PT-B skin was frequently observed in the dermis just below the epidermis (Fig 2E), and some fluorescence-labelled *P*. *aeruginosa* formed small colonies in the reticular dermis (Fig 2F and 2G), indicating the invasion of bacteria into the reticular dermis.

Notably, cluster formation of bacteria into nucleoids was observed in the epidermis and papillary dermis of all PT-B samples (Fig 2B and 2H). Inflammatory cells remarkably infiltrated in and around these clusters (Fig 2H and 2I). Within these clusters, intense bacterial signals were detected by immunohistochemistry and fluorescent labeling (Fig 2J–2L). These findings indicate the proliferation of *P*. *aeruginosa* within the proteolytic macerated skin, which could induce serious tissue damage from inside the skin.

### Healing process of inner tissue damage due to bacterial inoculation in proteolytic skin maceration

**Fig 3:**
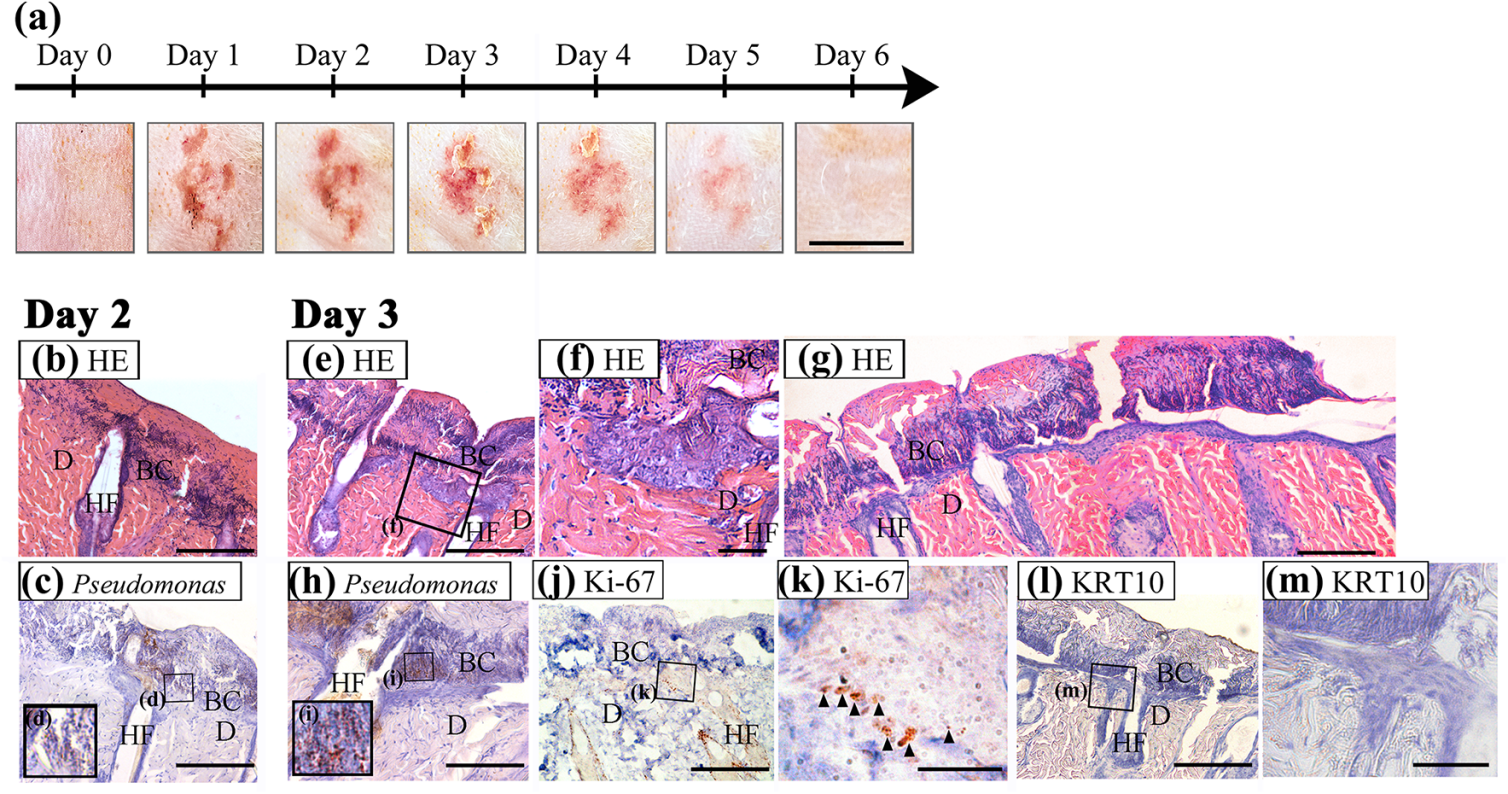
Healing process of tissue damage and bacterial cluster in proteolytic skin maceration. (a) Skin appearance was observed every day from day 1 to 6. The area of skin redness became larger, and redness got stronger over time until day 3. The redness faded after day 3 and returned to normal. Scale bar = 1 cm. Histological changes in proteolytic skin maceration were observed on day 2 (b, c) and day 3 (e-m). Thin sections were stained with HE (b, e, f, g), with immunohistochemistry for *Pseudomonas* species (c, d, h, i), with Ki 67 (j, k) and with keratin 10 (l, m). The boxed fields in (c), (e), (h), (j) and (l) are magnified in (d), (f), (i), (k) and (m), respectively. On day 3, the keratinocyte layer was elongated from the bulge region of hair follicles in the dermis deeper than the bacteria-invaded area (e). The alignment and stratification of cells like the epidermis were not observed in the keratinocyte layer (f). In this layer, many proliferative cells by Ki 67 immunoreaction were found (j, k), whereas no differentiated keratinocyte by keratin 10 immunoreaction was detected (l, m). Scale bar = 100 μm in (b), (c), (e), (g), (h), (j) and (l), and 20 μm in (f), (k) and (m); D, dermis; BC, bacterial cluster; HF, hair follicle.

To document the healing process after tissue damage and bacterial cluster formation on proteolytic macerated skin, we examined macroscopic skin appearance, histological changes, and bacterial localization in PT-B skin until the macroscopic healing of skin damage was observed. The findings are shown in Fig 3. The contralateral side of PT-B skin was also observed as the non-treated control. Neither macroscopic change nor histological change was found in NT skin.

Fig 3A shows macroscopic skin appearance. The area of skin redness became larger and stronger until 3 days post-inoculation. The redness gradually faded and natural colour was restored by 6 days post-inoculation.

Histology and the distribution of bacteria are shown in Fig 3B–3H. On day 2, bacterial clusters identified by HE staining (Fig 3B) and immunohistochemistry for *Pseudomonas* (Fig 3C) spread, and invaded deeper into the dermis. On day 3, which was the tipping point for a recovery in skin appearance, an epithelium-like layer was elongated from the bulge region of hair follicles in the dermis, and went deeper than the bacteria-invaded area (Fig 3E and 3F). In some area, this epithelium-like layer contacted the real epidermis (Fig 3G). The alignment and stratification of cells like those of the epidermis were not observed in this layer. This epithelium-like structure was completely negative for keratin 10, which is a marker of keratinocyte differentiation, and included abundant proliferative cells (Fig 3I–3L).

## Discussion

This study was conducted to reveal the histopathologies of macerated skin exposed to proteases and bacteria. This was the first attempt to reproduce and examine skin conditions accompanying fecal incontinence. Proteolytic skin maceration with bacteria inoculation induced considerable inner skin-tissue digestion, the healing process of which was similar to transepidermal elimination. These findings suggested that the histopathology of incontinence-associated skin lesions is quite different from that of contact dermatitis.

In PT skin, significantly higher levels of skin hydration and TEWL were observed than in NT skin, which indicated that our method induced skin maceration and impaired skin barrier function. In this study, proteolytic treatment increased TEWL to 4.7 times that of NT skin. In contrast, a previous study showed that TEWL increased to 2.3 times the untreated level in skin maceration of rat foot sole [9]. This difference suggests that proteolytic skin maceration results in a greater impairment of skin barrier function compared with normal saline. Moreover, Minematsu et al. [9] also reported that skin macerated by normal saline allowed transdermal penetration of macromolecules into the deeper layer of the dermis. Therefore, we speculate that proteolytic macerated skin has accelerated transdermal penetration of macromolecules. Our histological analysis of PT skin sections showed more severe tissue damage, compared with non-macerated skin with trypsin treatment, in which thickening and loosening of the corneal layer or hyperproliferation of all layers of the epidermis were observed [16, 27]. These results indicate that transdermally-penetrated proteases in proteolytic macerated skin cause tissue degradation from inside the skin, whereas protease-treated skin without maceration causes degradation from the skin surface.

In proteolytic macerated skin, bacteria invaded into the deeper layer of the dermis by bacterial colony formation, proliferation, and clustering. Colonization is meaningful for tissue damage, because colonization of bacteria promotes the expression of virulence factors such as proteases and lipases [28, 29]. Therefore, our observation of colonization and cluster formation in bacteria-loaded proteolytic skin maceration suggests that bacterial virulence factors contribute to tissue degradation from inside the skin. Bacterial clusters in our findings were similar to the lichenoid tissue reaction, which is defined as an infiltrate of inflammatory cells that fills the papillary dermis in a band-like fashion and occurs in skin disorders such as drug eruption or in the skin of patients with lupus erythematosus.

It has been recognized that skin exposed to urine and/or feces results in irritant contact dermatitis. The histopathology of irritant contact dermatitis is limited to the epidermis or dermal-epidermal junction [30]. Tissue damage and bacterial cluster formation in the dermis, which were found in PT-B skin, are out of the definitions of contact dermatitis.

In the healing process of PT-B, inner tissue damage and bacterial clusters may be divided from the normal dermis by the keratinocyte layer elongated from the hair follicle. This healing process of PT-B is similar to transepidermal elimination, which occurs spontaneously in certain skin disorders such as Kyrle disease and perforating folliculitis [31].

Conventionally, therapeutic intervention for incontinence-associated skin lesions is compliant with the treatment of contact dermatitis. However, findings in this study suggest that the histopathology of incontinence-associated skin lesions might be different from that of contact dermatitis. This suggests that we need to explore new skin care therapies according to the histopathology of incontinence-associated skin lesions.

Generalization of this new research to the healthcare field requires clinical study of the histopathology of proteolytic maceration in patients with fecal incontinence. To develop novel skin care techniques, further research is needed to reveal the effects of lipase from feces and bacteria on skin lesions. Furthermore, understanding the healing process more precisely will help develop effective therapeutic techniques for incontinence-associated skin lesions. Further analysis to identify the hair follicles by immunohistochemistry in the healing process will be required. These efforts will contribute to improving the quality of life of many patients suffering from skin lesions due to fecal incontinence.

## Conclusions

We reproduced macerated skin in rats using proteolytic macerated skin with bacterial inoculation as a model of skin exposed by fecal incontinence, and examined histopathological changes. Proteolytic skin maceration and bacterial inoculation induced inner tissue damage with bacteria-rich clusters, although outer skin appearance showed only skin flare. There is a possibility that the histopathology of skin lesions caused by feces differs from regular dermatitis. Advanced nursing care of macerated skin in patients with fecal incontinence can be required to prevent proteolytic skin maceration.
